# Prevalence of Illness Anxiety Disorder and Its Relation to Anxiety Among Medical Students in Karachi: A Cross‐Sectional Study

**DOI:** 10.1002/brb3.71430

**Published:** 2026-04-22

**Authors:** Wajiha Aftab, Hafsa FNU, Sheikh Abdul Qadir Jillani, Sahar Imtiaz, Muhammad Ahmed Zahoor, Sumia Fatima, Muddassir Khalid, Fred Segawa

**Affiliations:** ^1^ Department of Medicine Dow International Medical College Karachi Pakistan; ^2^ Department of Medicine Dow Medical College Karachi Pakistan; ^3^ Rawalpindi Medical University Rawalpindi Pakistan; ^4^ Department of Community Medicine Nishtar Medical University Multan Pakistan; ^5^ Makerere University College of Health Sciences Kampala Uganda

**Keywords:** anxiety (D001007), hypochondriasis (D006998), illness anxiety disorder, medical education (D004501), medical students (D013337), nosophobia (C000719212), psychological distress (D000079225)

## Abstract

**Background:**

Medical education is associated with high levels of stress that can intensify health‐related anxiety, referred to as illness anxiety disorder, medical student syndrome, or nosophobia. This phenomenon often occurs when medical students, after studying a particular illness, start to feel they are having its symptoms and believe they may have the disease. We aimed to determine the prevalence of illness anxiety disorder and its relationship with generalized anxiety among medical students in Karachi.

**Methods:**

This cross‐sectional study was carried out from February 2025 to May 2025 among MBBS students in Karachi. A pre‐validated structured questionnaire, comprising demographic details, the Short Health Anxiety Inventory (SHAI) scale, the Medical Students’ Disease (MSD) Perception Scale, and the MSD Distress Scale, was distributed among 300 students from first to fifth year.

**Results:**

Out of 300 students, significant health‐related anxiety (SHAI ≥ 18) was present in 16% (*n* = 48), and moderate to severe generalized anxiety (GAD‐7 score ≥ 10) was present in 28% (*n* = 83) of the students. The highest mean SHAI score was shown by fifth‐year students (12.31 ± 1.12), while the highest mean GAD‐7 scores were shown by second‐year students (7.25 ± 0.69). Gender and academic year did not have a statistically significant association with SHAI and GAD‐7 scores (*p* > 0.05). Moreover, the Pearson correlation test revealed a low positive but statistically significant correlation between SHAI and GAD‐7 scores (*r* = 0.462, *p* < 0.001). Linear regression analysis also revealed that the GAD‐7 score was a significant predictor of the SHAI score, accounting for 21.4% of the variance in the SHAI score (*β* = 0.462, 95% CI: 0.425–0.663, *p* < 0.001*).

**Conclusion:**

Health‐related anxiety and generalized anxiety are prevalent and correlated among the medical students of Karachi. The results of this study emphasize that we must target our mental health interventions to alleviate anxiety‐related distress in this population.

## Graphical abstract

Medical education may increase **health‐related anxiety (illness anxiety disorder)**, often referred to as **medical student syndrome**.

Cross‐sectional study (Feb–May 2025)

300 MBBS students (1st–5th year) – Karachi

Assessment tools:

- Short Health Anxiety Inventory (SHAI)
- GAD‐7 Anxiety Scale
- Medical Students’ Disease Perception and Distress Scales

Key Results

- Illness anxiety (SHAI ≥18): **16% (48 students)**
- Moderate–severe anxiety (GAD‐7 ≥10): **28% (83 students)**
- Highest SHAI: **5th year students**
- Highest GAD‐7: **2nd year students**
- **Positive correlation**: r = 0.462, p < 0.001

Illness anxiety and generalized anxiety are **common and significantly correlated** among medical students in Karachi, highlighting the **need for targeted mental health interventions**.

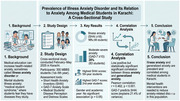

## 1 Introduction

High levels of stress are an inherent feature of medical education, as students encounter demanding academic requirements, clinical rotations, and the harsh realities of patient illnesses. This environment may intensify health‐related anxiety, frequently referred to as “medical student syndrome” or “nosophobia”—an irrational apprehension of contracting diseases that are the focus of their studies (Azuri et al. 2010; Hunter et al. 1964). Hypochondriasis, which is now classified as illness anxiety disorder (IAD) in the DSM‐5, entails an excessive fixation on the possibility of suffering from a serious illness, even in the face of medical reassurance (Bandyopadhyay et al. 2014).

In Pakistan, a study published in 2016 found that 11.9% of medical students suffered from illness anxiety disorder (Zahid et al. 2016). A meta‐analysis in China reported the pooled prevalence of illness anxiety disorder among health science students as 28% (Meng et al. 2019). Recent post‐pandemic data show significant differences in health‐related anxiety among medical students worldwide. In North Africa, studies from 2022 to 2024 reported health anxiety rates of 24.6% in Tunisia (Boussaid et al. 2025) and 15.7% in Egypt (Terra et al. 2024). In contrast, data from the United Arab Emirates indicated a lower prevalence of 9.3% according to the Short Health Anxiety Inventory (SHAI) (Abdel Aziz et al. 2023). During the peak of the COVID‐19 pandemic, anxiety linked to the virus reached 55% in some South Asian samples, with high rates of cyberchondria, including up to 50% of cases being severe (Sravani et al. 2022). In Europe, ongoing assessments revealed that 35.5% of medical students feared contracting the virus, while health anxiety remained notably higher among students than among practicing physicians (Najar et al. 2025). Previous studies suggest that medical students may exhibit higher levels of health anxiety compared with nonmedical students, attributable to heightened awareness of diseases and greater clinical exposure (Waterman and Weinman 2014). However, findings show considerable cultural variability, with specific studies indicating no substantial disparities in health anxiety between medical and nonmedical students (Szczurek et al. 2021). In Pakistan, there exists a paucity of research investigating illness anxiety disorder tendencies among medical students, despite the distinct stressors they encounter, such as geopolitical instability and a challenging 5‐year undergraduate curriculum that includes early clinical exposure (Valsraj et al. 2024).

This study aims to assess the prevalence of health‐related anxiety and how it relates to generalized anxiety among medical students in Karachi. We hypothesize that senior students will show significant health anxiety because they have more clinical exposure and that generalized anxiety will positively correlate with health anxiety across cohort studies, with no major gender differences.

## 2 Methods

This cross‐sectional study was conducted among MBBS students at Dow Medical College and Dow International Medical College in Karachi, Pakistan. Both institutions offer a 5‐year MBBS program, with the first 2 years primarily focused on preclinical studies, and the next 3 years involving clinical rotations alongside academic courses.

Medical students from first to final year enrolled in MBBS at Dow Medical College and Dow International Medical College, who were willing to participate and provide consent, were included in the study, whereas those students who did not provide consent or those who submitted incomplete questionnaires were excluded.

The sample size was calculated using OpenEpi version 3. By using the estimated prevalence of 11.9% found in a previous study conducted in Karachi (Zahid et al. 2016) and a confidence interval of 99%, the sample size was calculated to be 279. To account for potential nonresponders, 8% were added to the total sample size, making it 300. Participants were recruited using convenience sampling, in which individuals were selected based on their availability and willingness to participate. Questionnaires were distributed both electronically via WhatsApp groups and physically to students at both colleges.

A pre‐validated, structured questionnaire adapted from a previous study (Zahid et al. 2016) was used. It comprised four sections: (1) Demographic and Personal History—encompassed variables such as gender, academic year, history of medical consultations within the preceding 6 months, and the frequency of such consultations. (2) SHAI—a validated 18‐item instrument (validation studies report Cronbach's alpha coefficients between 0.87 and 0.95) designed to evaluate health‐related anxiety (Abramowitz et al. 2007). Items 1–14 constituted the primary section assessing illness anxiety disorder apprehensions, while Items 15–18 examined concerns regarding the negative ramifications of illness. Each item provided four response options (a–d), scored from 0 to 3. A threshold score of ≥ 18 on the main section was employed to delineate significant health anxiety, consistent with prior scholarly literature (Zahid et al. 2016). (3) MSD Perception and Distress Scales (Moss‐Morris and Petrie 2001)—evaluated the cognitive and emotional dimensions of health‐related anxiety. Each scale consisted of five items, with responses rated on a 5‐point Likert scale (1 = *Definitely false* to 5 = *Definitely true*). Cumulative scores were computed independently for both scales. (4) Generalized Anxiety Disorder Scale (GAD‐7)—a 7‐item screening instrument (it has shown excellent internal consistency with Cronbach's alpha reported as 0.92) aimed at assessing generalized anxiety over the preceding 2 weeks, with responses scored from 0 to 3 (Spitzer et al. 2006). A total score out of 21 was derived for each respondent.

The questionnaire was distributed over a period of 4 months (February 2025–May 2025). The participation in the study was voluntary. Written informed consent was obtained from all participants through a consent statement included at the beginning of the questionnaire, which participants were obligated to read and accept prior to proceeding with the study. No personal identifiers were documented; each questionnaire was assigned a code, and data confidentiality was vigilantly upheld throughout the process. This research followed the ethical guidelines and received approval from the Ethics Committee of Rawalpindi Medical University (Ethical code: M‐14‐16) on November 5, 2024.

Data analysis was done using IBM SPSS Statistics version 26. Descriptive statistics were employed to summarize the demographic characteristics and scale scores. Categorical variables were expressed as frequencies and percentages, whereas numerical variables were defined as means and standard deviations. Chi‐square tests, *t*‐tests, and ANOVA were used to determine associations between health anxiety (based on SHAI cutoff) and variables such as gender, academic year, recent doctor visits, and anxiety scores (GAD‐7). Binary logistic regression was conducted to identify predictors of significant health anxiety. A *p*‐value of less than 0.05 was considered statistically significant.

## 3 Results

### 3.1 Demographic Characteristics of Participants

A total of 300 medical students participated in this study. Of these, 195 (65%) were female, and 105 (35%) were male. The distribution across academic years was as follows: First year, 48 (16%); second year, 64 (21.33%); third year, 104 (34.67%); fourth year, 55 (18.33%); and fifth year, 29 (9.67%).

When asked about doctor visits during the past 6 months, 148 (49.33%) reported visiting a doctor, while 152 (51.67%) did not. Among those who visited, 45.94% visited once, 47.29% visited two to three times, 2.70% visited four to five times, and 4.05% visited more than five times (see Table 1).

**TABLE 1:** Demographic characteristics of participants.

Variable	Categories	Frequency (*n*)	Percentage (%)
Gender	Male	105	35
	Female	195	65
Year of study	First year	48	16
	Second year	64	21.33
	Third year	104	34.67
	Fourth year	55	18.33
	Fifth year	29	9.67
Doctor visits	Yes	148	49.33
(In the last 6 months)	No	152	51.67
Frequency of visits	1	68	45.94
	2–3	70	47.29
	4–5	4	2.70
	> 5	6	4.05

### 3.2 Descriptive Statistics of Key Scales by Year of Study

The mean SHAI score was relatively stable across all academic years, with a slight increase noted in the fifth year (mean ± SD = 12.31 ± 1.122). MSD Perception scores were also similar across years, with fifth‐year students showing the highest mean (mean ± SD = 15.48 ± 0.719). The GAD‐7 scores ranged from 6.62 to 7.25, with the highest anxiety reported by second‐year students (mean ± SD = 7.25 ± 0.694) (see Table 2).

**TABLE 2:** Mean and standard deviation of SHAI, MSD Perception, MSD Distress, and GAD‐7 scores by year of study.

Scale	Year of study	Mean ± SD	Min–Max
SHAI Score (Short Health Anxiety Inventory)	First year	11.27 ± 1.01	1–36
	Second year	11.17 ± 0.77	2–34
	Third year	11.29 ± 0.61	1–37
	Fourth year	11.78 ± 1.05	0–38
	Fifth year	12.31 ± 1.12	2–27
MSD Perception	First year	14.13 ± 0.56	6–25
	Second year	14.13 ± 0.65	5–25
	Third year	14.16 ± 0.45	5–25
	Fourth year	13.36 ± 0.63	5–22
	Fifth year	15.48 ± 0.72	8–24
MSD Distress	First year	12.77 ± 0.68	6–25
	Second year	13.45 ± 0.75	5–25
	Third year	12.77 ± 0.52	5–25
	Fourth year	12.11 ± 0.68	5–23
	Fifth year	13.17 ± 0.94	5–23
GAD‐7 Score	First year	7.00 ± 0.80	0–21
	Second year	7.25 ± 0.69	0–21
	Third year	6.63 ± 0.56	0–21
	Fourth year	6.84 ± 0.76	0–21
	Fifth year	6.62 ± 1.08	0–21

### 3.3 Prevalence of Health Anxiety and Generalized Anxiety

**FIGURE 1:**
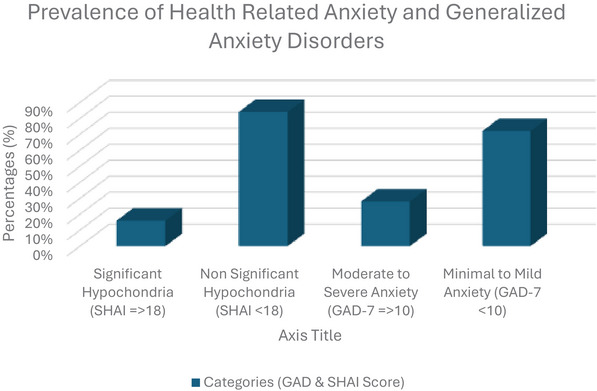
Prevalence of health‐related anxiety and generalized anxiety among participants (*N* = 300). Health anxiety was measured using the Short Health Anxiety Inventory (SHAI) and categorized as significant (≥ 18) or nonsignificant (< 18). Generalized anxiety was assessed using GAD‐7, categorized as moderate to severe (≥ 10) or minimal to mild (< 10). Bars represent the percentage of participants in each category.

A notable prevalence of health anxiety and generalized anxiety was observed among the students. Out of 300, 48 students (16%) achieved scores of ≥ 18 on the SHAI, reflecting clinically significant health anxiety. Moreover, 83 students (28%) had moderate (scores 10–14) to severe (scores 15–21) levels of generalized anxiety based on the GAD‐7 scale (see Figure 1).

### 3.4 Association Between Health Anxiety and Generalized Anxiety

**FIGURE 2:**
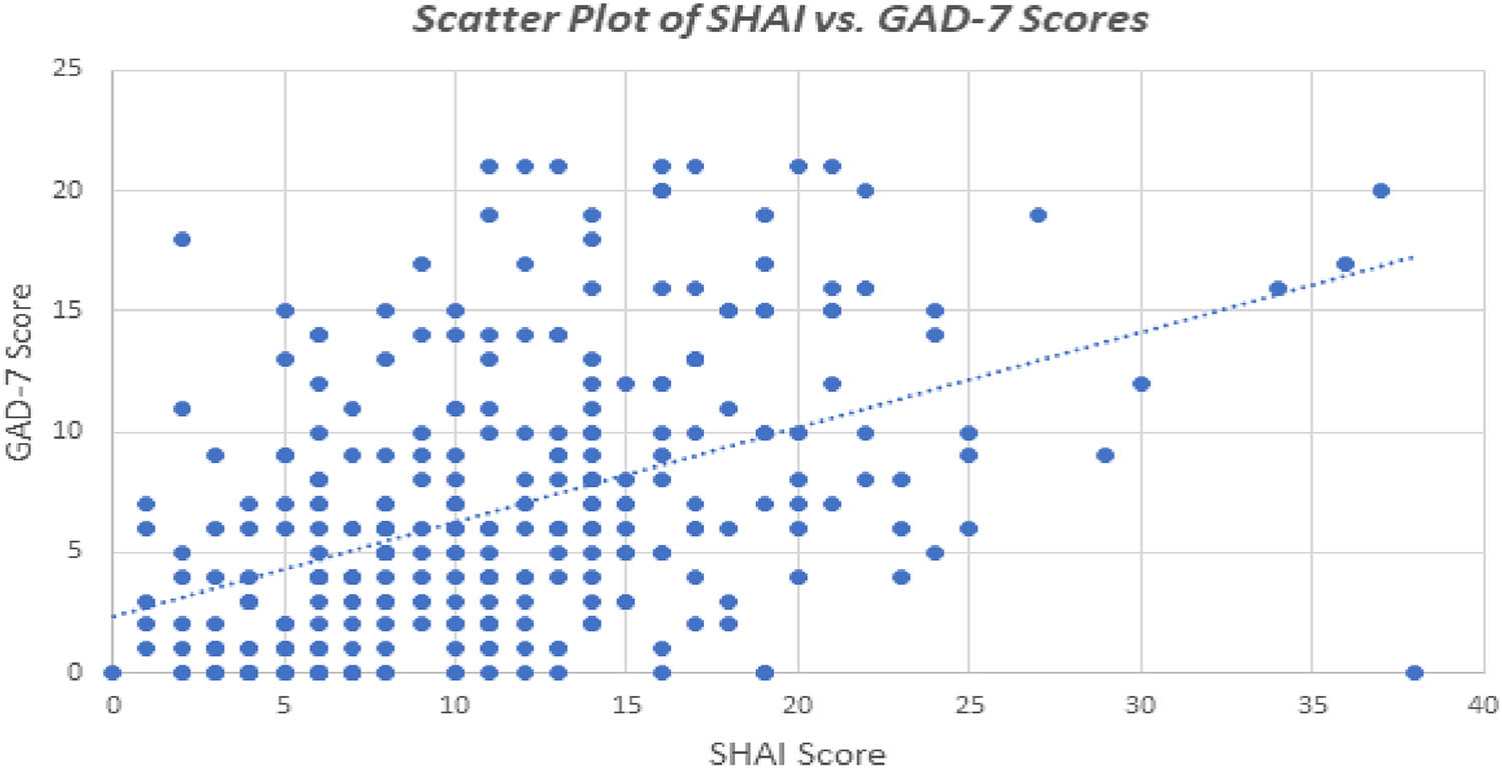
Scatter plot showing the correlation between Short Health Anxiety Inventory (SHAI) scores (0–37) and Generalized Anxiety Disorder‐7 (GAD‐7) scores (0–21) among participants (*N* = 300). Each point represents an individual participant; the dotted line indicates the linear regression trend, demonstrating the correlation between SHAI and GAD‐7 scores.

A Pearson correlation test revealed a low, but statistically significant, positive correlation between SHAI and GAD‐7 scores (*r* = 0.462, *p* < 0.001). This suggests that as generalized anxiety levels increase, there is a corresponding increase in health anxiety among medical students (see Figure 2).

### 3.5 Comparison of Health Anxiety and Generalized Anxiety Across Groups

*By Gender*

An independent *t*‐test showed no statistically significant difference in SHAI scores between males and females (*p* = 0.982). Similarly, GAD‐7 scores did not differ significantly across genders (*p* = 0.143) (see Table 3).

**TABLE 3:** Comparison of SHAI and GAD‐7 scores by gender.

Gender	SHAI (mean ± SD)	*p*‐value	GAD‐7 (mean ± SD)	*p*‐value
Male	11.44 ± 7.17	0.982	6.21 ± 5.35	0.143
Female	11.46 ± 6.28		7.21 ± 5.72	

*By Year of Study*

A one‐way ANOVA revealed no statistically significant difference in SHAI scores (*p* = 0.933) or GAD‐7 scores (*p* = 0.966) across different years of study (see Table 4).

**TABLE 4:** Comparison of SHAI and GAD‐7 scores by year of study.

Year of study	SHAI (mean ± SD)	*p*‐value	GAD‐7 (mean ± SD)	*p*‐value
First year	11.27 ± 1.01	0.933	7.00 ± 0.80	0.966
Second year	11.17 ± 0.77		7.25 ± 0.69	
Third year	11.29 ± 0.61		6.63 ± 0.56	
Fourth year	11.78 ± 1.05		6.84 ± 0.76	
Fifth year	12.31 ± 1.12		6.62 ± 1.08	

### 3.6 Linear Regression Analysis

**FIGURE 3:**
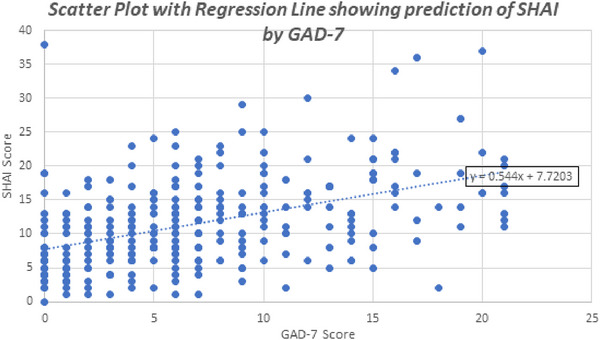
Scatter plot with regression line illustrating the prediction of SHAI scores by GAD‐7 scores among 300 participants. SHAI (0–37) assesses health anxiety, and GAD‐7 (0–21) measures generalized anxiety. The regression line (*y* = 0.544*x* + 7.72) indicates a positive relationship.

A linear regression analysis was conducted to predict SHAI scores based on GAD‐7 scores. The model was statistically significant (F(1,298) = 81.035, *p* < 0.001) and explained 21.4% of the variance in SHAI scores (*R* ^2^ = 0.214). The GAD‐7 score was a significant predictor of health anxiety (*β* = 0.462, 95% CI: 0.425–0.663, *p* < 0.001*) (see Figure 3 and Table 5).

**TABLE 5:** Linear regression predicting SHAI from GAD‐7 scores.

Predictor	Beta coefficient	95% CI	*p*‐value	*R* ^2^
GAD‐7 Score	0.462	0.425‐0.663	< 0.001*	0.214

## 4 Discussion

This study aimed to investigate the prevalence of health‐related anxiety among medical students. Our study is unique because it specifically addresses health‐related anxiety among medical students in Pakistan, a topic that has been largely overlooked in existing research (Zahid et al. 2016). Other similar studies from Pakistan have focused more specifically on anxiety and depression among medical students, rather than specifically on health‐related anxiety, highlighting the lack of studies directly addressing this topic (Hussain et al. 2025; Mufarrih et al. 2019; Kamran et al. 2023). Globally, medical students experience substantial academic stress; however, it is essential to note that Pakistani medical students have a very different educational, cultural, and social background compared to those in Western/developed nations. In Pakistan, a 5‐year undergraduate program with clinical exposure begins in the third year (Pakistan Medical & Dental Council 2024). This is quite different from the curricula in developed countries. In addition, the geopolitical instability of Karachi also has a toll on mental well‐being (Valsraj et al. 2024). Therefore, this study was conducted to determine whether health‐related anxiety is a significant stressor among medical students in Karachi, Pakistan. Our study included a total of 300 students, with 65% being females and 35% being males across all five academic years. Determining the prevalence of health‐related anxiety in medical students in Pakistan is essential to gauge this phenomenon and formulate mechanisms to minimize it.

The results indicated that SHAI scores reflected a high level of consistency across all the clinical years, with a slight increase found in the fifth year, possibly due to increased clinical exposure and academic pressures. Interestingly, the maximum GAD‐7 scores were found in second‐year students, with a decline observed stepwise through to the final year. This reflects a probable peak of general anxiety at the beginning of clinical training when the students are first exposed to patient care and clinical rotations, and cope with a heavy load of theoretical coursework. The level of consistency reported in SHAI scores, being highest in the final year, may be a reflection of an enhanced experience with clinical cases, and this may lead to worsening of health‐related anxiety, particularly among the health‐anxiety‐prone students.

The correlation between health anxiety and generalized anxiety was also examined. Pearson correlation analysis revealed a small but statistically significant positive correlation. Linear regression analysis revealed that GAD‐7 was significantly associated with SHAI, accounting for 21.4% of the variance in the outcome. These findings are consistent with earlier work highlighting shared cognitive‐affective processes between generalized anxiety and anxiety disorders specific to health (Azuri et al. 2010; Moss‐Morris and Petrie 2001). This highlights the importance of using integrated psychological approaches to help medical students deal with both specific and general stressors.

Despite the observed trends, there were no statistically significant differences in SHAI or GAD‐7 scores based on gender and academic year, which contrasts with international studies that often find higher anxiety levels in female students (Mohamed et al. 2024; Quek et al. 2019; Dahlin et al. 2005). Societal perceptions of mental health, stigma around psychological vulnerability, and gender norms related to emotional expression may affect how students report anxiety symptoms. Furthermore, male students may underreport due to cultural expectations of resilience, while female students may normalize stress as an inherent part of medical training. All these factors may play a part in explaining these differences.

The finding that a large proportion of the students who had seen a doctor within the last 6 months reported multiple visits suggests a pattern of health‐related anxiety, characterized by frequent reassurance‐seeking behavior. Existing studies have illustrated that individuals with health anxiety present with patterns of overuse of healthcare services, such as repeated consultations and investigative procedures in pursuit of reassurance (Sunderland et al. 2013; Hannah et al. 2023; Norbye et al. 2022). Not only does such a pattern foretell the mental well‐being of students, but it also adds to the strain on healthcare resources, which is of particular concern in under‐resourced settings, such as in Pakistan.

In a broader perspective, the findings underscore the vital importance of developing organized, culturally responsive mental health programs in medical schools. Since generalized anxiety plays a significant role in health anxiety, stress reduction interventions, normalization of illness‐related thoughts, and resilience may help reduce the prevalence of health‐related anxiety. Interventions like mindfulness‐based stress reduction (MBSR), educational interventions in typical psychological reactions faced by medical students, and mentorship programs led by faculty members can act as buffers against increasing levels of anxiety.

The finding that fifth‐year students, with the most clinical experience, also achieved the highest scores in both the SHAI and MSD Perception is interesting. It is plausible that greater exposure over time to patients' distress, combined with increased academic pressures, exam‐related stress, and uncertainty over career paths, might exacerbate previously held health concerns. Although some studies indicate that clinical exposure can reduce health anxiety, the type and quantity of that exposure, that is, in high‐stress clinical environments, may have opposite effects. Final‐year students are commonly exposed to rotations through high‐mortality departments, such as oncology, critical care, and internal medicine, which may work to reinforce health anxieties, particularly among those who are already anxious.

Moreover, the cognitive and emotional distress factors of health anxiety, as measured on the MSD Perception and MSD Distress scales, had varied trends. In other words, MSD Perception scores were highest among final‐year students, indicating a high level of cognitive concern or preoccupation with illness. Conversely, the MSD Distress scores were highest among second‐year students, suggesting that emotional distress is most significant during the initial years of academic pursuit. This disparity observed lends support to the dual‐component model of health anxiety proposed by Moss‐Morris and Petrie (2001), suggesting the possibility of utilizing specific intervention methods targeting cognitive distortions and emotional distress separately.

### 4.1 Strengths and Limitations

Methodologically, this study has several strengths, including a sample size of 300 students, which is statistically robust, representation of all academic years, and the use of standardized psychometric instruments like the SHAI and GAD‐7, which have been validated in previous research. However, certain limitations should be acknowledged. First, the cross‐sectional design provides only a single time‐point assessment and precludes causal inference or evaluation of temporal trends in anxiety across medical training. Second, reliance on self‐report questionnaires introduces the possibility of response bias, including social desirability bias, underreporting in stigmatizing environments, and potential recall bias. Nonresponse bias may also limit representativeness if students experiencing higher or lower anxiety levels were less likely to participate. Third, the absence of a nonmedical control group limits attribution of the observed anxiety patterns specifically to medical training, particularly given evidence that students in other competitive academic settings may also report elevated health anxiety (Mofatteh 2020; Mirza et al. 2021). Additionally, the use of international SHAI cutoff scores may not fully account for cultural variation in symptom expression. Future research should focus on cross‐cultural validation within the Pakistani population and consider deriving regional cutoff values using structured clinical interviews or focus groups. Cross‐validation with instruments like the Health Anxiety Questionnaire (Lucock and Morley 1996) and the Whitley Index (Conradt et al. 2006) could further enhance diagnostic specificity.

## 5 Conclusion

This study shows that medical students in Karachi often experience health‐related anxiety, which is linked to higher levels of general anxiety. This finding underscores the necessity of integrating mental health policies within medical institutions, including the implementation of early psychological screening protocols and stress reduction interventions. Furthermore, the incorporation of mentorship and mindfulness techniques into medical curricula could potentially bolster students' enduring resilience and their capacity to navigate the emotional challenges inherent in clinical training.

## Author Contributions

**Wajiha Aftab**: conceptualization, literature review, writing – original draft. **FNU Hafsa**: writing – original draft. **Sheikh Abdul Qadir Jillani**: writing – original draft. **Sahar Imtiaz**: methodology, formal analysis, investigation. **Muhammad Ahmed Zahoor**: visualization, validation, figure, and table creation. **Muddassir Khalid**: supervision, project administration, writing – review and editing. **Sumia Fatima**: supervision, project administration, writing – review and editing. **Fred Segawa**: final revision.

## Funding

The authors have nothing to report.

## Conflicts of Interest

The authors declare no conflicts of interest.

## Data Availability

Data available on request from the authors.
